# Exploiting Injury-Induced Peripheral Opioid Receptor Changes in Novel Analgesic Development for Chronic Pain

**DOI:** 10.3389/fpain.2022.883164

**Published:** 2022-04-26

**Authors:** Craig T. Hartrick

**Affiliations:** Caventure Drug Discovery, Bloomfield Hills, MI, United States

**Keywords:** pain receptors, injury induced plasticity, drug development, biomarkers, novel analgesics

## Introduction

The process of novel analgesic drug development, from ideation to sustainable marketing, has many steps: each with multiple challenges. Successful evolution to safe, effective, and economically viable clinical use requires a series of positive outcomes in both scientific and business arenas. Failure at any point can result in the abandonment of further development of the new molecular entity (NME). In fact, most early analgesic development approaches end well before regulatory approval and marketing for clinical use. Only 2% of NMEs for pain transitioned to Food and Drug Administration (FDA) approval compared with 10% for all diseases from 2007 through 2017 (1).

When one considers the enormity of the problems associated with the most potent currently available analgesics, opioids, the urgency of the unmet medical need cannot be overstated (2). Still non-opioid options, especially in the US, are shrinking rather than growing. The removal of marketed cyclooxygenase-2 (COX-2) inhibitors from the US market (3) and other jurisdictions, and the failure of approval for newer COX-2 agents (4) have made this situation even more acute. With few NMEs coming online, development has focused on recycling already approved traditional opioids with novel delivery systems (5, 6) or abuse deterrent formulations (7). A biased mu opioid agonist, oliceridine, is among the few NMEs to reach regulatory approval in recent years (8). While it does provide an improved safety profile with respect to respiratory depression (9), like the other mu opioids, this remains a risk. Moreover, mu opioids, as centrally acting agents, carry with them the additional risk of addiction (10). According to the Centers for Disease Control and Prevention (CDC), 1 out of 4 persons receiving prescription opioids suffer from addiction, most having experienced their first opioid in the form of a prescription from a physician (11).

Expanding the physicians' armamentarium to address moderate-to-severe chronic pain beyond archetypal mu opioids is an urgent unmet need. Chronic pain, by some estimates, affects nearly one-third of the population worldwide (12). Yet it is important to note that failure to address this therapeutic gap has not been the result of apathy or lack of effort on the part of pharma or the scientific community. Numerous NMEs have shown great promise in animal models only to fail in clinical trials. Some failures may have conceivably been attributed to failure to reach effective concentrations sufficient for target engagement due to inappropriately low dosing or suboptimal formulation. Historically, there were undoubtedly other failures that might well have been avoided by applying the same rigor to preclinical investigations that has been recognized as good practice in clinical trials for many years (13). Despite considerable advances directed toward both the conduct and reporting of preclinical trials (14–16), difficulties in translation to the human pain state remain.

The thesis presented here is that there are at least two more major knowledge gaps that have hampered successful development of analgesic NMEs. Urgently needed are: (1) better preclinical pain models and pain measurement methods (or methods to eliminate animal models altogether), and (2) a better understanding of the alterations in pain pathways associated with chronic pain states. These two areas requiring improved understanding are interrelated. While often assumed that specific behavioral observations and responses to various stimuli reflect pain in preclinical models, this is often far from certain. However, since preclinical models aim to emulate specific chronic pain conditions, ensuring similar underlying mechanisms are present in both the preclinical and the clinical models strengthens confidence in the ability to monitor analgesic response in animals unable to provide self-report.

## Preclinical Pain Model Translation

The current regulatory process involves preclinical small animal efficacy testing (usually rodents), followed by both small and larger animal (typically canine) safety testing, before Phase I clinical trials on healthy volunteers. Efficacy and safety investigations in humans who suffer from the specific pain indication(s) of interest begin much later in Phase II and III clinical trials after many millions of dollars have already been spent (17). Failure at this late stage results in a dampening effect on investment and on the enthusiasm for the entire process. The inclusion of Proof-of-Concept (PoC) and Proof-of-Activity (PoA) studies early in clinical development might help identify biomarkers for better candidate selection in subsequent Phase II and II trials, thus improving outcomes (18). The use of biomarkers, especially in clinical phase I and II have been estimated to potentially double the chance of successful FDA approval (19).

Biomarkers can take the form of genetic, physical or laboratory testing, neuroimaging, or other observations that can be correlated with diagnostic, prognostic, or therapeutic responses (20). Often mentioned in reference to clinical chronic pain conditions is the use of quantitative sensory testing (QST). QST is particularly useful in chronic pain states affecting sensory perception thresholds and tolerability (e.g., hyperalgesia, allodynia, hyperpathia). Neuroimaging, while challenging logistically, could also be of value in both preclinical and clinical models. Genetic testing is appealing, but interspecies differences limit generalizability. Skin biopsy is feasible in both preclinical and clinical models. In conditions associated with peripheral small-fiber neuronal changes, intra-epidermal nerve fiber density examination has been used for diagnostic, pharmacodynamic response, predictive, and prognostic purposes (21, 22). Situations where injury-induced changes in opioid receptor density or behavior are expressed in affected tissues in the periphery lend themselves to this latter PoC approach. Biopsy to directly assay peripheral opioid receptor density has been performed in the preclinical setting (23). For clinical study, less invasive approaches may be desirable. Indirect or surrogate studies assessing the response to local challenge with chemically (e.g., capsaicin), thermally (e.g., UV: ultraviolet radiation) or electrically evoked stimuli can be useful in first-in-human drug studies to document activity (PoA) (24), and potentially in patient selection for later clinical studies to differentiate candidates with preexisting inflammation-induced hyperalgesic skin from less susceptible candidates in select clinical settings. Accordingly, a more effective preclinical process, incorporating relevant biomarkers, should also result in greater success. However, unless and until methods are developed that will be accepted by regulatory authorities that obviate the need for animal studies, proactively attempting to improve the translation from lower species to humans remains critical.

This first issue, interspecies differences, has been concisely described elsewhere (25). Briefly, humans are not large rats, and unfortunately even rats are not just large mice. Expecting rodents to express behaviors analogous to complex pain characteristics associated with chronic pain in humans, such as the emotional/affective and suffering components, is not reasonable given the anatomic differences in forebrain structure. These authors also make the important point that chronic pain expression, as assessed by behavioral changes in rodents, is further complicated by the fact that rodents are prey animals. Visible evidence of vulnerability in a prey animal is not conducive to survival and thus the reluctance to show signs of weakness following injury is more adaptive than stoic. Altered burrowing behavior may be more indicative of distress than other observations such as weight-bearing, gait abnormalities or other spontaneous behaviors presumed to relate to pain (26, 27). The use of small non-human primates (28, 29), if accepted by regulatory bodies, could potentially improve translation, especially if naturally occurring pain conditions provided an ethical opportunity for study. While much can be gleaned from human *ex-vivo* human tissue regarding mechanism (30, 31), response to a novel treatment can only be ascertained *in vivo*. Moreover, the clinical human population, far from a specific strain of animal selected to create uniformity in preclinical testing, are more like “wild type” animals. The diverse responses to analgesics reflect this fact, again emphasizing the importance of biomarkers in subject selection in clinical trials, as well as the use of biomarkers in the earlier preclinical studies (22).

It is well known that the “standard” preclinical pain models used to emulate various human chronic pain conditions produce idiosyncratic responses (such as altered licking, vocalization, facial expressions, weight-bearing, feeding, social interaction, ambulation, rearing, burrowing, and conditioned place preference) that are assumed to relate to the pain experience (32). These models imperfectly match clinical conditions with respect to the inciting injuries, longitudinal time course following injury, and genetic predisposition (33). Similarly, typical interventions imperfectly match duration of treatment, as most preclinical efficacy trials involve a single or a limited number of repeat doses. Nor can they, as limited duration studies, account for subsequent time-dependent adaptation, including the development of tolerance to intervention. Longer-term studies are performed in safety testing; applying this same strategy to efficacy testing could potentially improve success in the clinical setting.

By way of example, the types of injuries that are commonly used to study potential treatments for neuropathic pain include placing ligatures around the sciatic nerve (chronic constriction injury; CCI), ligating a portion of the sciatic nerve (partial nerve injury; PNI), ligation of spinal nerves contributing to the sciatic nerve (spinal nerve ligation; SNL), and ligation of two of the three terminal branches of the sciatic nerve (spared nerve injury; SNI). These models develop on a different time courses, last different periods of time, result in differing degrees of evoked behaviors such as mechanical hyperalgesia, mechanical allodynia, and thermal hyperalgesia, and exhibit differences in spontaneous behaviors as well (34). A model focusing on the initiation of neuroinflammation without overt nerve injury has been proposed whereby the sciatic nerve is surrounded by a cuff that bathes the nerve in zymosan (Sciatic inflammatory neuritis; SIN). However, it still involves surgical incision (itself a confounding injury) and implantation of the foreign body (35). A variation on this approach whereby the zymosan is injected percutaneously with no surgical incision or implanted foreign body other than the zymosan has been described (36). These latter two approaches have not been widely accepted, but arguably are less contrived. Additionally, contralateral effects in the non-surgical percutaneous model, as in the clinical condition, were largely absent, in contrast to the dose-dependent contralateral effects in SIN and the less prominent but still consistently reproducible contralateral effects noted in CCI (37). Regardless, clinical neuropathic pain conditions only rarely involve injuries due to suture or section of all or part of the sciatic nerve, its roots, or its branches. The models do, however, allow for examination of features that may relate to symptoms experienced in some patients. Back translating from human to animal by identifying patient phenotypes based on QST or other biomarkers has been suggested as a “precision medicine” method to improve preclinical pain model selection (38). Absent better models, this approach seems completely reasonable.

## Peripheral Opioid Receptor Changes

Just as humans are not large rodents, somewhat analogously chronic pain is not persistent acute pain. Chronic pain often involves altered pain pathways. Long-term potentiation, microglial activation, synaptic pruning and other mechanisms contribute to the neuroplasticity that can cause pain to become intransigent and independent of the initial inciting injury. In these cases, chronic pain becomes a disease in and of itself (39, 40).

Given the importance of mu opioid receptors (MOR) within principal pain pathways in the central nervous system (brain and spinal cord), up- or down-regulation of MORs in number or function impacts the ability of traditional mu opioids to modulate chronic pain states. Furthermore, in some cases, alterations in MOR activity in chronic pain states appear to relate to specific insults. Several common chronic pain states are recognized to be relatively unresponsive to mu opioid agonists and characteristically difficult to treat, including bone cancer, fibromyalgia, and neuropathic pain.

MORs are down-regulated in bone cancer (41). Alternatively, multiple non-opioid pain pathways are activated, including enhanced involvement of inflammatory mediators such as bradykinin (42). In fibromyalgia, investigators have described reduced central availability of MORs (43). Neuropathic pain states are similarly characterized by a shift away from mu-opioid dominated pathways to noradrenergic pathways (44). The lack of efficacy in these situations contributes to inappropriate and futile dose escalation. Consequently, these phenotypic shifts, along with alterations due to the act of mu agonist administration inducing tolerance and addiction, are among the reasons that traditional mu opioids have not been shown to be consistently superior to non-opioid analgesics in chronic pain states (45, 46).

In contrast, injuries resulting in peripheral inflammation, at later stages, eventually induce migration of MORs into peripheral tissues (47, 48), offering the opportunity for mu opioid agonists to have distinct analgesic effect apart from their action in the central nervous system. Analgesics, including mu opioids, that remain in the periphery avoid central MOR-mediated sedative effects, respiratory depression, and the potential for addiction. Unfortunately, mu opioid agonists can themselves induce microglial activation that induces hyperalgesia, lowers pain thresholds, and induces a primed microglial phenotype that persists even after opioid discontinuation, thus worsening rather than alleviating chronic pain (49).

Inflammation also induces changes in non-mu opioid receptor activity in the periphery. Kappa opioid receptors (KOR) are constitutively present peripherally and may participate in anti-inflammatory induced analgesia (50). However, even absent inflammation, peripheral KORs actively mediate analgesia (51). Yet in the presence of inflammation, kappa opioid-mediated analgesia is enhanced. This enhancement likely results, at least in part, from synergistic action on delta opioid receptors (DOR). The DOR, also constitutively present in the periphery, is normally quiescent, being functionally impeded by G Protein-Coupled Receptor Kinase-2 (GRK2). In the presence of inflammation, the DOR receptor becomes active. Proinflammatory bradykinin stimulates GRK2 movement away from DOR and onto Raf Kinase Inhibitory Protein (RKIP). Protein kinase C (PKC)-dependent RKIP phosphorylation associated with the binding of bradykinin (BK) induces this GRK2 sequestration, restoring DOR functionality in sensory neurons (52). Additionally, KORs and DORs form heterodimers. The activity of these heterodimers has been demonstrated in peripheral sensory neurons and the allosteric interaction between the kappa and delta components of the heterodimers is thought to contribute to the enhancement of kappa-mediated analgesia by delta agonists (53).

## Discussion

Adaptation to both injury and exposure to exogenous toxins can be essential for survival. Drugs, as exogenous substances, can be considered potentially toxic assaults to be pharmacokinetically metabolized and eliminated. Perhaps less well appreciated are altered pathways that pharmacodynamically minimize the drug's effects though the induction of tachyphylaxis, tolerance, or other mechanisms. These processes can become even more significant with repeated administration, as is required in chronic conditions. Thus, over time these adaptations may severely impair the efficacy of the administered drug. Agents that appear to be effective in short-term preclinical models may subsequently fail with longer-term administration clinically. In contrast, when the altered pathways responsible for these effects are understood, they can be exploited to improve drug efficacy in specific conditions. The phenotypic shift in neuropathic pain, away from opioid-mediated pathways in favor of noradrenergic pathways, as previously mentioned, provides just such an opportunity. Further, because adrenergic receptors are up-regulated in peripheral sensory neurons (54), they are readily accessible for study. This well-known and long studied observation could have been exploited as a useful biomarker for screening and recruiting susceptible candidates for inclusion in clinical trials, as well as optimizing the selection of preclinical models for detailed study.

Another NME among the very few novel analgesics to be FDA approved in recent decades is tapentadol. It is one of the multiple potential analgesics demonstrating significant noradrenergic effects, suggesting utility in neuropathic pain states (55). Mechanistically, tapentadol's relatively modest analgesic activity attributed to action at the MOR is greatly magnified by synergistic noradrenergic reuptake inhibition (56). While the effect on descending central pathways may be largely responsible for its non-opioid analgesic effect, had initial preclinical and early clinical trials focused on noradrenergic receptor upregulation, as a peripherally accessible biomarker, the development process might well have been accelerated Ultimately, tapentadol extended release was approved for neuropathic pain associated with diabetic peripheral neuropathy (57, 58).

Many chronic pain conditions, including neuropathic pain, involve the initiation of inflammatory pathways, albeit to varying degrees. One NME, CAV1001, has demonstrated efficacy in multiple preclinical models (59). CAV1001, as a dual-acting, peripherally restricted kappa/delta opioid agonist, was more effective in reducing inflammatory-induced hyperalgesia in mice by an order of magnitude when compared on an equimolar basis to a peripherally restricted pure kappa opioid agonist. This synergy is consistent with DOR becoming active in the inflammatory state and consequent potential involvement of DOR/KOR heterodimers. Moreover, it was shown to be relatively more potent in preclinical models known to have greater involvement of the inflammatory cascade (59). The use of a biomarker designed to identify heterodimers of DOR/KOR and their behavior following injury in the selection of candidates for subsequent clinical trials could greatly improve the odds for successful clinical development of this NME. Absent immunostaining or other practical technique to directly assess peripheral receptor density in sensory neurons from skin biopsy, indirect evidence for inflammation-induced hyperalgesic responses could be used.

UV-evoked inflammatory hyperalgesia has been used as an early biomarker for efficacy in early human studies on volunteers. Although the technique causes no significant injury, it does result in the local elaboration of multiple inflammatory mediators (24, 60). This model has been used experimentally in human volunteers to confirm efficacy for multiple drugs, including opioids (61). Importantly, the technique has also been successfully used in preclinical translational models (62). Additional application of laser algesimetry, where a CO_2_ laser stimulus is used to generate somatosensory evoked potentials, can add an objective measure of efficacy that has been used in healthy volunteers with UV-induced inflammatory hyperalgesic skin (24) and might be useful in clinical trial subject screening. Numerous drugs have been evaluated with this technique including mu-, kappa- and the mixed-opioid agonists tramadol, tapentadol, and pentazocine.

While the aforementioned factors are important, they illustrate just two among many explanations as to why most seemingly promising preclinical drugs go on to fail in clinical trials. The examples given also reinforce the concept that no single analgesic can be expected to be useful for all chronic pain states, and that mechanism based multimodal approaches will remain best practice for the foreseeable future. Novel analgesic development should focus on a customized, personalized, biomarker driven approach that fully considers not only very specific mechanisms of action, but also the underlying pathophysiology as it exists within each genetically and epigenetically unique chronic pain patient.

## Author Contributions

The author confirms being the sole contributor of this work and has approved it for publication.

## Author Disclaimer

The opinions expressed are not those of any institution and are attributed solely to CH.

## Conflict of Interest

CH is a co-founder and stockholder in Caventure Drug Discovery.

## Publisher's Note

All claims expressed in this article are solely those of the authors and do not necessarily represent those of their affiliated organizations, or those of the publisher, the editors and the reviewers. Any product that may be evaluated in this article, or claim that may be made by its manufacturer, is not guaranteed or endorsed by the publisher.
